# Genetic diversity analysis and development of molecular markers for the identification of largemouth bass (*Micropterus salmoides* L.) based on whole-genome re-sequencing

**DOI:** 10.3389/fgene.2022.936610

**Published:** 2022-08-29

**Authors:** Jinxing Du, Shengjie Li, Jiaqi Shao, Hongmei Song, Peng Jiang, Caixia Lei, Junjie Bai, Linqiang Han

**Affiliations:** ^1^ Key Laboratory of Tropical and Subtropical Fishery Resources Application and Cultivation, Ministry of Agriculture and Rural Affairs, Pearl River Fisheries Research Institute, Chinese Academy of Fishery Sciences, Guangzhou, China; ^2^ Guangdong Liangshi Aquatic Seed Industry Co Ltd, Foshan, China

**Keywords:** largemouth bass, genetic diversity, germplasm identification, SNP, INDEL

## Abstract

Largemouth bass (*Micropterus salmoides* L.) is generally considered to comprise two subspecies, Florida bass (*M. floridanus*) and Northern Largemouth bass (*M. salmoides*), which have biological characteristic differences because of their geographical distribution. In this study, whole-genome re-sequencing was performed among 10 Florida and 10 Northern largemouth bass, respectively. In total, 999,793 SNPs and 227,797 InDels were finally identified, and 507,401 SNPs (50.75%) and 116,213 InDels (51.01%) were successfully mapped to annotated 18,629 genes and 14,060 genes, respectively. KEGG classification indicated that most of these genes were focused on the pathways including signal transduction, transport and catabolism, and endocrine system. Genetic diversity analysis indicated that Florida largemouth bass had higher genetic diversity than Northern largemouth bass, indicating that the germplasm quality of Northern largemouth bass had markedly reduced in China. To examine the accuracies of the identified markers, 23 SNPs and eight InDels (the insertions or deletions of more than 45 bp) were randomly selected and detected among Florida largemouth bass, Northern largemouth bass, and their F1 hybrids. The detection efficiencies of all the markers were higher than 95%; nineteen SNPs and three InDels could accurately distinguish the two subspecies and their F1 hybrids with 100% efficiencies. Moreover, the three InDel markers could clearly distinguish the two subspecies and their F1 hybrids with a PCR-based agarose gel electrophoresis. In conclusion, our study established a simple PCR-based method for the germplasm identification of largemouth bass, which will be useful in the germplasm protection, management, and hybridization breeding of largemouth bass.

## Introduction

Largemouth bass (*Micropterus salmoides* L.) is native to freshwater lakes and rivers in North America. It is considered to comprise two subspecies (Bailey and Hubbs, 1949), the Florida largemouth bass (*M. floridanus*, FB) distributed in the Florida peninsula, and the Northern largemouth bass (*M. salmoides*, NB) distributed in most central and eastern parts of America, northeast Mexico, and southeast areas of Canada (Maceina and Murphy, 1992). With its worldwide introduction in the 1970s, it was introduced from Taiwan to mainland China in 1983 and has become an economically cultured fish in China with an output of 620,000 tons in 2020. Our teams collected mainly largemouth bass populations in different areas in China and re-imported the wild FB and NB populations from America in 2010 (Cai et al., 2012). Through morphological traits, microsatellite molecular markers (Fan et al., 2009), and DNA fingerprinting (Fan et al., 2012), we confirmed that the cultured largemouth bass in China belongs to the Northern subspecies. In the past two decades, two new varieties of largemouth bass have been selectively bred by our team based on the NB population: “Youlu No.1” bred in 2011 and “Youlu No.3” bred in 2019 (Bai and Li, 2019). However, the genetic diversity of cultured largemouth bass has been markedly reduced during the breeding process (Bai et al., 2008; Fan et al., 2019).

Hybridization, as one of the important methods for fish breeding, can effectively transfer good parental traits and increase the genetic variation of offspring (Hulata 2001; Lou 2007). Currently, several hybridization studies and the comparison of the genetic structures of the hybrids and their parents have been reported in largemouth bass. In the reciprocal cross of cultured NB and imported FB in 2010, the observed heterozygosity (*Ho*), expected heterozygosity (*He*), and polymorphic information content (PIC) were all highest in FB ♀ × NB ♂, which were 0.849, 0.639, and 0.571, respectively (Cai et al., 2012). In the reciprocal cross of “Youlu No.1” and NB imported in 2010, the *Ho*, *He*, and PIC values were all highest in “Youlu No.1” ♀ × NB ♂, which was 0.729, 0.553, and 0.454, respectively (Zhou et al., 2020). Recently, in the genetic diversity of F1 progeny on cross species of FB, NB, and “Youlu No.3”, the *Ho*, *He*, and PIC were all highest in “Youlu No.3” ♀ × FB ♂, which was 0.559, 0.600, and 0.521, respectively (Wang et al., 2020). These results indicated that hybridization has an obvious effect on the improvement of genetic diversity in largemouth bass.

Accurately distinguishing the hybrids and their parents is the first step in hybridization breeding of largemouth bass. Because the morphological traits of the hybrids are midway between FB and NB, it makes classification of largemouth bass difficult based on morphology alone (Rogers et al., 2006). During the last several decades, molecular genetic markers, such as mitochondrial DNA (mtDNA) analysis with restriction fragment length polymorphism (RFLP) (Nedbal and Philipp, 1994), random amplified polymorphic DNA (RAPD) (Williams et al., 1998), specific sites of the isoenzyme (Philipp et al., 1983), and microsatellite molecular markers (Lutz-Carrillo et al., 2006; Lutz-Carrillo et al., 2008; Seyoum et al., 2013), have been applied to a variety of genetic studies of largemouth bass (Austin et al., 2012; Hargrove et al., 2019). With the development of sequencing technology, the third generation of DNA markers is based on single nucleotide polymorphisms (SNPs) and insertion/deletion (InDel), have been widely used in the germplasm identification in plants and animals (Zhang et al., 2017; Guo. et al., 2019; Thongda et al., 2019; Zhao et al., 2019). Currently, gene-linked SNP markers have been discovered (Li et al., 2015) and applied to the classification of largemouth bass (Hargrove et al., 2019), while the InDel markers are less reported. Compared with the requirement of special equipment systems for SNP detection, the detection of InDel technology is user-friendly and low-cost (Guo et al., 2019), which has high application values in the germplasm classification of largemouth bass.

The obtainment of the largemouth bass reference genome (GenBank: GCA_019677235.1) provides an advantage for the discovery of SNP and InDel markers between NB and FB. In this study, 10 “pure” FB and 10 “pure” NB were selected and sequenced with a whole-genome re-sequencing technique. Then, the SNP and InDel markers were screened and annotated according to the reference genome of largemouth bass. In addition, genetic diversity analysis was conducted based on the high-quality SNPs, and a series of SNP and InDel markers were randomly selected and detected among FB, NB, and their F1 hybrids. Our study provided a PCR-based method for the germplasm identification of largemouth bass, which would be useful in the protection, management, and hybridization breeding of largemouth bass.

## Materials and methods

### Sample collection

Largemouth bass were collected from the Pearl River Fisheries Research Institute, Chinese Academy of Fishery Sciences (Guangzhou, China). FB was originally imported from America in 2010, and NB was the new variety of largemouth bass “Youlu No.3” bred in 2019. Identifications of these fish were conducted based on a subset of microsatellite primers from Malloy et al. (2000) and Lutz-Carrillo et al. (2008). Then, the fin tissues used for whole-genome re-sequencing were collected from 10 FB (N1-N10) and 10 NB (F1-F10), respectively. All the fin tissues were stored at −80°C until DNA extraction.

### DNA extraction and Re-sequencing

For each sample, genomic DNA was extracted from 0.1 g fin tissue using a modified CTAB method (Stewart and Via, 1993). The extracted DNA samples were quantified using a NanoDrop 2000 spectrophotometer (NanoDrop Technologies, Wilmington, DE, United States) and their quality was assessed through 0.8% agarose gel electrophoresis. Library construction for re-sequencing was prepared with 1.0 μg of starting total DNA and processed using the VAHTS universal DNA Library Prep Kit for MGI (Vazyme, Nanjing, China) following the manufacturer’s recommendations. Paired-end libraries with an insertion size of 350 bp were constructed for 10 NB and 10 FB, respectively, and index codes were added to attribute sequences to each sample. The library quantification and size were measured using the Qubit 3.0 Fluorometer (Life Technologies, Carlsbad, CA, United States) and the Bioanalyzer 2,100 system (Agilent Technologies, CA, United States). Subsequently, sequencing was performed on an MGI-SEQ 2000 platform by Frasergen Bioinformatics Co., Ltd. (Wuhan, China).

### Detection and annotation of SNP and InDels

After removing adapter sequences and low-quality reads, the clean reads were further rechecked for quality based on the following criteria: consecutive bases on the ends with base quality <20, read length <50 bp, and the singletons were also removed. High-quality sequences were aligned and mapped to the reference genome of largemouth bass using a BWA program (v0.7.17) (Li and Durbin, 2009) with default settings. The sequencing depth and coverage compared to the reference genome were calculated based on the alignment results using SAMtool software (Li et al., 2009).

SNPs and InDels were called using the Genome Analysis Toolkit (GATK) Haplotype Caller (McKenna et al., 2010). To reduce the error rate of calling variations, SNPs were filtered by variant filtration tools with the following threshold: QD < 2.0, FS > 60.0, MQ < 40.0, MQRankSum < −12.5, and ReadPosRankSum < −8.0. Meanwhile, InDels were filtered by GATK with the recommended threshold: QD < 2.0, FS > 200.0, and ReadPosRankSum < −20.0. The mutational positions and genomic regions, were assessed using ANNOVAR (v2019). The genome-wide distribution of mutations was plotted using Circos software (v0.69). Finally, the functional annotation of the genes with mutations were predicted by Nr, SwissProt, GO, KOG, and KEGG databases with DIAMOND (ver.0.9.22.123) software.

### Genetic diversity analysis

All the SNPs that passed filtering in the population were used to construct a phylogenetic tree using TreeBeST (http://treesoft.sourceforge.net/treebest.shtml/) (Vilella et al., 2009). The final phylogenetic tree was plotted using iTOL (http:/itol.embl.de). The principal component analysis was performed using PLINK v1.07 software with default parameters (Yang et al., 2011). Population structure clustering was analyzed using Admixture (v1.3.0) with K setting from 2 to 10. The K value with minimum cross-validation error was chosen the best population structure (Yang et al., 2011). Linkage disequilibrium (LD) was calculated using PopLDdecay (v3.30) (Zhang et al., 2019), and the LD decay was calculated based on the squared correlation coefficient (*r* ^2^) values between the two SNPs and the physical distance between the two SNPs. Nucleotide diversity (*π*) and inbreeding coefficient (F) were calculated using VCFtolls (v.0.1.13) (Danecek et al., 2011).

### Experimental validation of SNP and InDel markers

In total, 130 largemouth bass came from three populations were used to validate the accuracies of candidate SNP and InDel markers: 1) 20 FB and 20 NB re-imported from America in 2010; 2) 30 FB progenies of the 2010 re-imported FB bred in 2020, and 30 NB progenies of the “Youlu three” bred in 2020; 3) 30 F_1_ hybrids (NF1 -NF30) bred in 2020, which were obtained by “Youlu No.3” ♀ × re-imported FB in 2010 ♂. Total DNA was extracted from fin tissues using the standard phenol-chloroform procedure and DNA integrity was examined using agarose gel electrophoresis and quantity was determined using an Agilent 2,100 Bioanalyzer (Agilent, Shanghai, China).

The 30 NB, 30 FB, and 30 NF were used for the SNP detection. The polymorphisms of 23 randomly selected SNPs were detected with the Snapshot method. Briefly, primers were designed according to the largemouth bass genome sequences of these SNPs, respectively (Supplementary Table S1). Then, multiplex PCR amplification was performed with 1 μl DNA, 1 μl forward primer, and 1 μl reverse primer (10 pM), 7.5 μl Premix Taq™ (Takara, #RR901A), and 4.5 μl ddH_2_O. The PCR procedure was as followed: 94°C for 3 min, followed by 35 cycles of 94°C for 15 s, 58°C for 15 s, 72°C for 30 s, and finally, 72°C for 4 min. Then, the PCR production was purified with ExoI and FastAP. The reaction mixture was 3 μl PCR production, 0.2 μl ExoI, 0.8 μl FastAP, 0.7 μl ExoI buffer, and 3.3 μl ddH_2_O, respectively. The PCR procedure was as followed: 37°C for 15 min and 80°C for 15 min. Then, the extension reaction was performed with 2 μl purified PCR production, 1 μl Snapshot Ready ReactionMix, 1 μl extension primer (10 pM), and 3 μl ddH_2_O. The procedure was as follows: 96°C for 1 min, followed by 30 cycles of 96°C for 10 s, 52°C for 5 s, and 60°C for 30 s. Then, 10 μl Hidi formamide (Applied Biosystems, Foster City, United States) was added into 1 μl extension production and treated in the ice. Then, the detection of DNA polymorphisms was sequenced with an ABI3730XL Sequencer (Applied Biosystems, Foster City, CA).

To develop the InDel markers for the germplasm identification of largemouth bass, eight InDels were randomly selected from the identified InDels, with a size number >45 bp of the insertions or deletions bases in each of the InDel makers. These InDel markers were detected in the aforementioned NB, FB, and NF individuals. qPCR was performed in a 20 μl reaction mixture including 10 μl of Premix Taq™ (Takara, #RR901A), 0.5 μl of each primer (10 μm), 1 μl of DNA, and 8 μl of ddH_2_O. The PCR procedure was as follows: 94°C for 5 min, followed by 35 cycles of 94°C for 30 s, 58°C for 30 s, 72°C for 30 s, and finally, 72°C for 10 min. Then, electrophoresis on a 2.0% agarose gel was used to detect the sizes of PCR amplification products.

## Results

### Analysis of genome re-sequencing data

A total of 619,675,540 and 633,811,124 paired-end clean reads were generated from 10 NB to 10 FB, respectively, which had an average coverage depth of approximately 10× to largemouth bass reference genome (*Micropterus salmoides*). The overall mapping rate was 97.33% for NB and 95.57% for FB, respectively, with an average of 96.45%. Moreover, for NB and FB, 95.37 and 94.03% paired-end reads, and 0.09 and 0.17% single-end reads were mapped to the reference chromosomes of the largemouth bass genome, indicating the high quality of the sequencing data. In addition, nucleotide statistics on the assembled scaffolds showed that the GC content is nearly 40% (Table 1).

**TABLE 1:** Summary of the re-sequencing results of NB and FB.

Basic information	NB	FB
Total clean reads	619,675,540	633,811,124
Total clean base(bp)	92,025,014,273	94,176,077,761
Length	150	150
Average Q20	97.52	97.81
Average properly mapped (%)	95.37%	94.03%
Average singletons mapped (%)	0.09%	0.17%
Average map ratio (%)	99.51	99.67
Average depth (x)	10.31	10.40
Average cover ratio (%)	97.33	95.57
Average GC content (%)	39.99	40.00

### Genomic distribution and annotation of SNPs and InDels in largemouth bass

**FIGURE 1:**
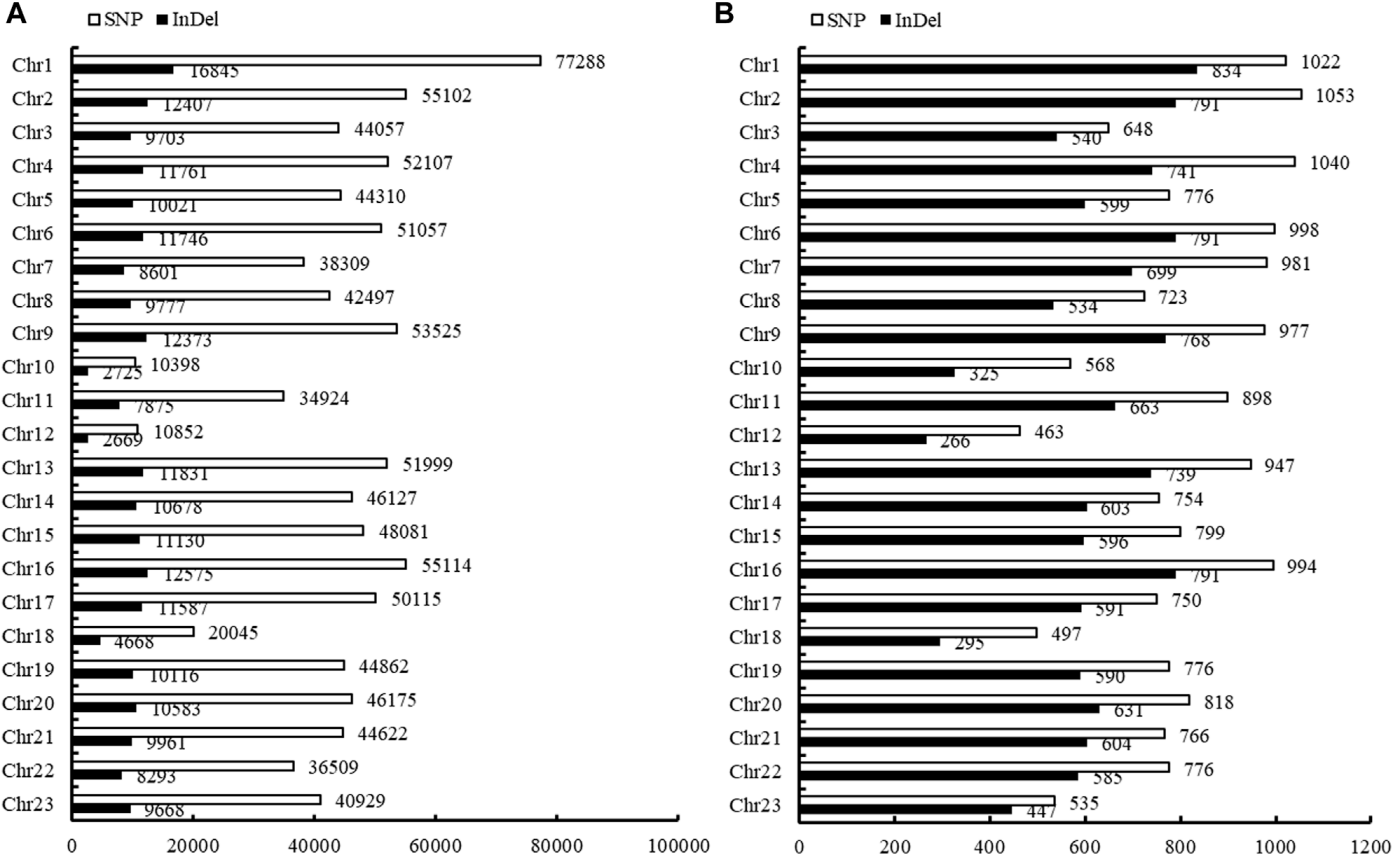
Disruption of SNP and InDels in the genome and genes of Largemouth bass. **(A)** Disruption of SNP and InDels in each chromosome of the largemouth bass. **(B)** Annotation of the SNPs and InDels in the corresponding genes of the largemouth bass.

In total, 999,793 filtered SNPs were finally identified between NB and FB. These SNPs were disrupted across all the 23 chromosomes (Chr), and varied from 10,398 on Chr10 to 77,288 on Chr1 (Figure 1A). The densities of SNPs in the largemouth bass genome were estimated at 0.81 per kilobase (kb) each. A total of 507,401 SNPs (50.75%) were successfully mapped to the genome sequences of annotated 18,630 genes (a total of 23,901 genes in the genome) (Supplementary Table S2). The functional characterization of genes with the polymorphic SNPs was disrupted across 23 chromosomes of largemouth bass, which varied from 1,053 on Chr2 to 463 on Chr12 (Figure 1B).

**FIGURE 2:**
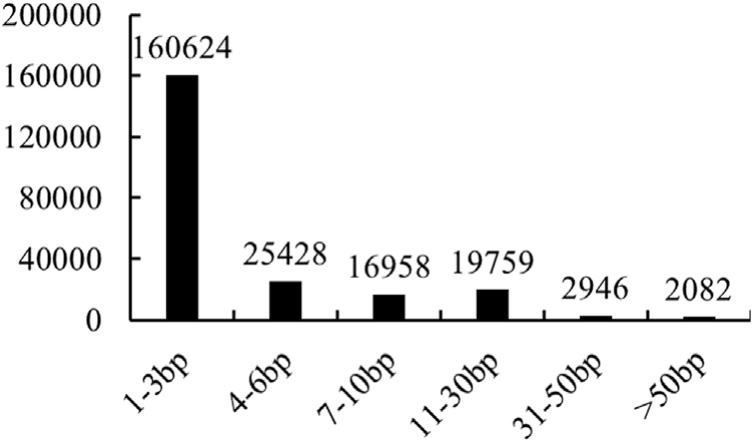
Summary of InDels between NB and FB.

In total, 227,797 filtered InDels were finally identified between NB and FB. These InDels varied from 2,669 on Chr12 to 16,845 on Chr1 (Figure 1A). The densities of InDel in the largemouth bass genome were estimated at 3.82 per kilobase (kb) each. The majority of InDels were small and ranged from 1 to 3 bp (70.51%), and InDels longer than 50 bp had the smallest proportion (0.91%) (Figure 2). A total of 16,213 InDels (51.01%) were successfully mapped to the genome sequences of the annotated 14,061 genes (Supplementary Table S3), which varied from 834 on Chr1 to 266 on Chr12 (Figure 1B).

**FIGURE 3:**
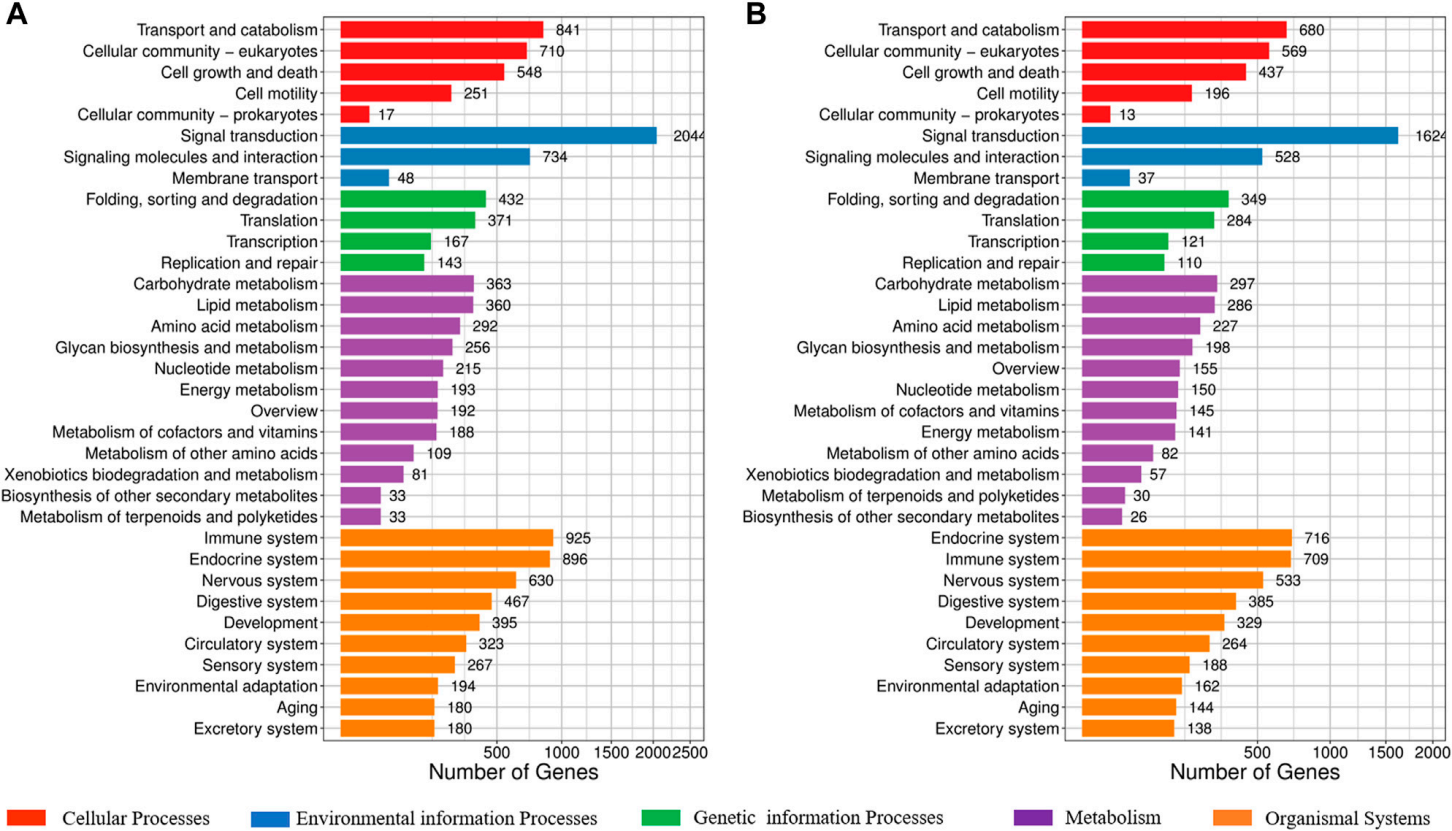
KEGG classification of SNPs and InDels in the potential biology process in the largemouth bass. **(A)** KEGG classification of SNPs in the potential biology process. **(B)** KEGG classification of InDels in the potential biology process.

KEGG classification suggested that these SNPs and InDel markers are widely involved in transport and catabolism, and the cellular community of cellular processes; signal transduction, signaling molecules, and interaction of environmental information processes; folding, sorting and degradation, and transcription of genetic information processes; carbohydrate metabolism and lipid metabolism of metabolism; immune system and endocrine system of organismal systems (Figures 3A,B, Supplementary Tables S4, S5). Specially, signal transduction process related genes consisted of most polymorphic SNPs and InDels.

### Genetic diversity analysis of FB and NB

**FIGURE 4:**
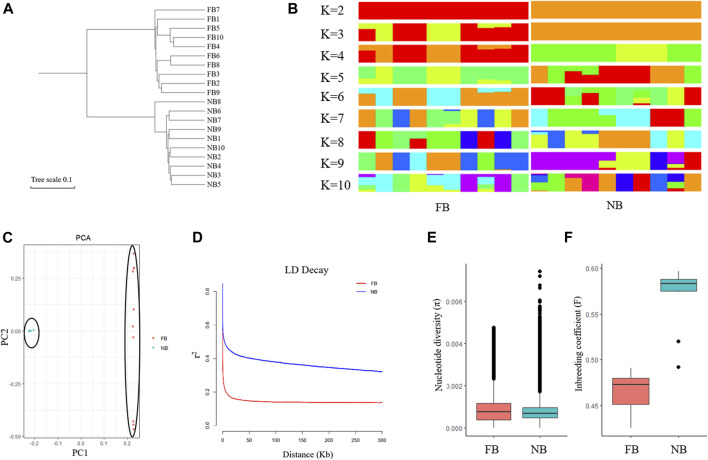
Genetic diversity analysis among 10 NB and 10 FB. **(A)** The phylogenetic tree analysis of 10 NB and 10 FB. **(B)** Population genetic structure analysis of 10 NB and 10 FB. **(C)** Principal component analysis (PCA) of10 NB and 10 FB. **(D)** Linkage disequilibrium (LD) analysis of 10 NB and 10 FB. **(E)** Nucleotide diversity (*π*) of 10 NB and 10 FB. **(F)** Inbreeding coefficient **(F)** of 10 NB and 10 FB.

To explore the relationships between the 10 NB and 10 FB, a neighbor-joining phylogenetic tree was constructed using all the filtered SNPs. The phylogenetic tree classified the 20 LMB into two groups: the 10 FB were clustered into one group and the 10 NB clustered into another group (Figure 4A). A population genetic structure analysis was performed based on the high-quality SNPs. We employed 5-fold cross-validation to infer the number of ancestral populations (Figure 4B; Supplementary Figure S1). At K = 2, NB showed a strong genetic differentiation from FB. At K = 3, the population structure of FB was complex, especially when the K value was higher, suggesting a higher genetic diversity in FB (Figure 4B).

Principal component analysis (PCA) was used to further confirm the relationship between the 10 NB and 10 FB. As shown in the principal component plot for the first two principal components, which accounted for 60.24% of the total variation observed in 20 largemouth bass. PC1 explained 55.71% of the overall variation and separated 20 largemouth bass into two groups named NB and FB, and PC2 explained 4.53% of the overall variation (Figure 4C). The LD decay rate in FB was faster than that in NB (Figure 4D). Moreover, the nucleotide diversity (*π*) of FB was higher than that of NB, while the inbreeding coefficient (F) of FB was lower than that of NB. Taken together, our results indicated that the NB population experienced a longer breeding process.

### Validation of SNP and InDel polymorphisms in FB, NB, and their F1 progenies

To test the efficiency of the identified SNPs, a total of 23 randomly selected SNPs were conducted in 30 NB, 30 FB, and their 30 F1 progenies (NF), with one on each chromosome. The results indicated that all the 23 SNPs performed good polymorphisms and 19 SNPs (82.60%) could distinguish these fish with 100% efficiencies (Table 2; Supplementary Table S6). The accuracies of the other four SNPs ranged from 96.67 to 97.78%, respectively.

**TABLE 2:** Genotypes and annotation of 23 randomly selected SNPs in 30 NB, 30 FB, and 30 NF individuals, respectively.

Number/disruption	Genotype			Annotated gene or related region
	NB (number)	FB(number)	NF(number)	
SNP1/Chr1	AA (30)	GG (30)	AG (30)	Intergenic region
SNP2/Chr2	TT (30)	AA (30)	AT (30)	Son of seven less homolog 1
SNP3/Chr3	GG (30)	AA (30)	AG (30)	Intergenic region
SNP4/Chr4	GG (30)	AA (30)	AG (30)	Leucine-rich repeat N-terminal domain
SNP5/Chr5	TT (30)	GG (30)	GT (30)	5-Formyltetrahydrofolate cyclo-ligase
SNP6/Chr6	AA (30)	CC (30)	AC (30)	Ubiquitin-conjugating enzyme E2 L3b
SNP7/Chr7	TT (30)	CC (27)	GT (30)	Epidermal growth factor-like domain
SNP8/Chr8	TT (28)	CC (30)	GT (30)	Nuclear apoptosis inducing factor 1
SNP9/Chr9	CC (30)	AA (30)	AC (30)	Intergenic region
SNP10/Chr10	GG (30)	TT (30)	GT (30)	Prohibition
SNP11/Chr11	CC (30)	TT (30)	CT (30)	Synaptotagmin XIa
SNP12/Chr12	CC (30)	TT (30)	CT (30)	Intergenic region
SNP13/Chr13	GG (30)	AA (30)	AG (30)	Intergenic region
SNP14/Chr14	GG (30)	AA (30)	AG (30)	Solute carrier family 35 member
SNP15/Chr15	AA (30)	TT (30)	AT (30)	Peptide methionine sulfoxide reductase MsrA
SNP16/Chr16	TT (30)	AA (30)	AT (30)	Intergenic region
SNP17/Chr17	TT (30)	AA (30)	AT (30)	Intergenic region
SNP18/Chr18	GG (30)	TT (30)	TT (30)	Intergenic region
SNP19/Chr19	CC (30)	AA (30)	AC (30)	Microtubule-actin cross-linking factor 1
SNP20/Chr20	TT (28)	CC (30)	GT (30)	Holocarboxylase synthetase
SNP21/Chr21	AA (30)	TT (30)	AT (30)	Intergenic region
SNP22/Chr22	AA (30)	GG (30)	AG (30)	Ankyrin 3b
SNP23/Chr23	AA (28)	TT (30)	AT (30)	Podocalyxin-like protein

**FIGURE 5:**
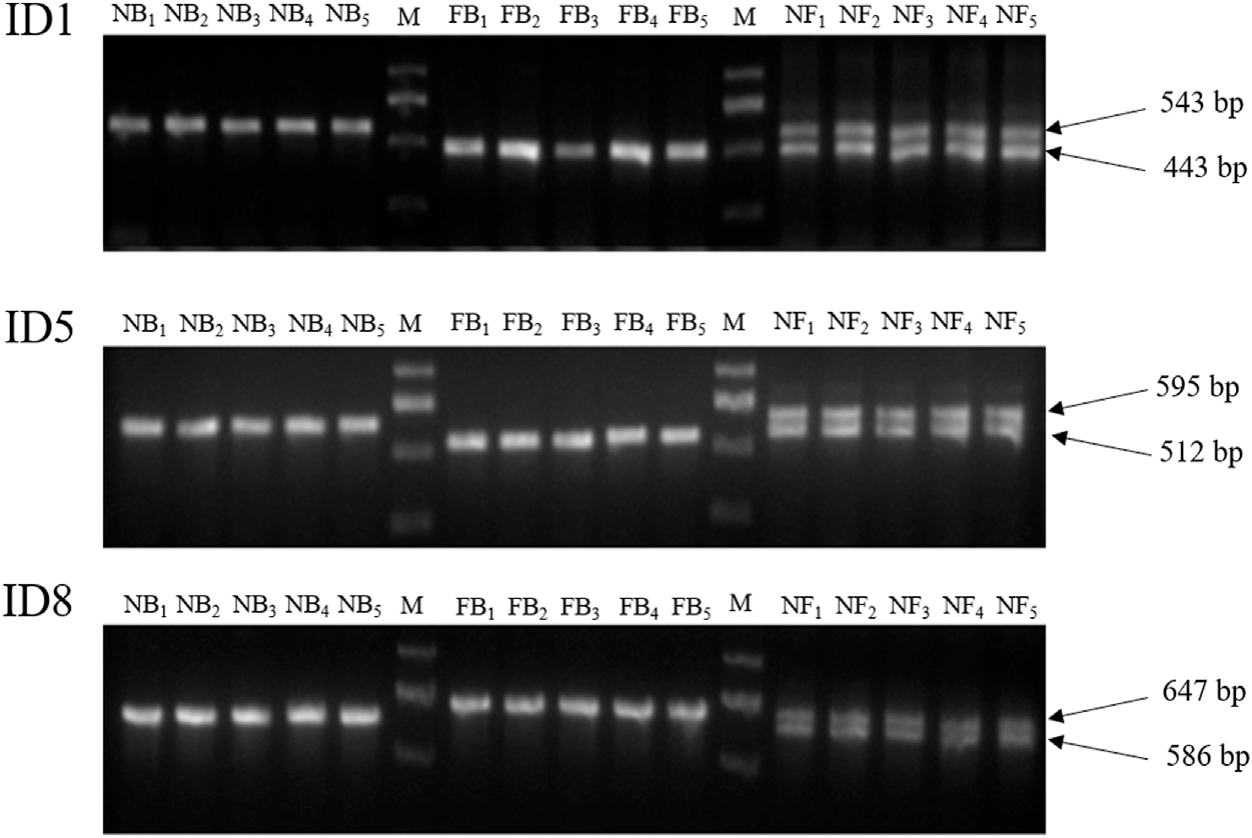
PCR amplification results of ID1, ID5, and ID8 in 5 NB, five FB, and five NF individuals, respectively. M, DNA Marker DL 2000. NB, Northern largemouth bass. FB, Florida largemouth bass. NF, the F1 hybrid of the Northern largemouth bass ♀ × Florida largemouth bass ♂.

In order to develop a PCR-based method for the germplasm identification of largemouth bass, a total of eight InDel markers were selected randomly from the aforementioned Indels, which were longer than 45 bp (Table 3). After PCR amplification with specific primers among 50 NB, 50 FB, and 30 NF, the PCR production were detected with 2% agarose gel electrophoresis, and only three InDel markers (ID1, ID5, and ID8) could accurately identify the NB, FB, and NF with 100% accuracies, with one different length stripe for NB and FB, respectively, and two stripes for the hybrids (Figure 5; Supplementary Figure S2). In addition, the efficiencies of other five InDel markers ranged from 96.15 to 97.69%, respectively.

**TABLE 3:** Primers and annotation of eight selected InDels in largemouth bass.

Indel location	Insertion/deletion size	Primer (5′-3′)	Annotated gene or related region
ID1/Chr17	+100	F: ACA​TTC​AGC​CCT​CTT​GAC​CG	Intergenic region
		R: GAC​ACG​GGG​AGA​TCA​TGC​AA	
ID2/Chr2	+90	F: CCT​TTG​TTA​ACC​TGC​CCC​CT	Intergenic region
		R: GTA​GTC​ATG​GGA​CCA​TCC​CC	
ID3/Chr3	+46	F: GCA​TCG​TTT​CCA​CAG​GTG​TC	Intergenic region
		R: GCA​GCT​TCC​AAT​GCA​ACT​GTA	
ID4/Chr6	+87	F: TCA​CGC​CAC​ATC​CAG​GTA​AG	Intergenic region
		R: TGC​CAT​AGG​TAA​CTC​CCC​AGT	
ID5/Chr20	+83	F: CGT​GTC​AGC​TAA​CTA​CAC​CTG​A	Intergenic region
		R: ATA​CTG​CCC​CGC​AAA​GGA​AA	
ID6/Chr5	−84	F: GTC​AAC​CGG​TGA​ACA​CAA​CG	Neural-cadherin-like
		R: ACG​TTA​TCA​GCA​CTG​TGC​CA	
ID7/Chr17	−51	F: AGG​GAG​AAA​CCT​CAT​TGG​GC	Intergenic region
		R: TTG​CTG​GCA​TCC​TCC​ATA​GC	
ID8/Chr23	−61	F: CAC​CAG​CCT​GCA​GGT​AAG​AA	Intergenic region
		R: CTT​CCA​ACC​ACA​CAA​GGT​CAG	

“+” and “-” mean insert and deletion in NB, in comparison with FB, respectively.

## Discussion

### Genome variation between FB and NB largemouth bass

To better understand the genetic variation in largemouth bass subspecies, 10 NB and 10 FB were selected for whole-genome re-sequencing. With the guidance of the largemouth bass reference genome, 999,793 SNPs were finally identified, which was much more than that in the previous studies, without the largemouth bass reference genome. For example, 3,674 SNPs were discovered through the transcriptomes analysis between NB and FB (Li et al., 2015); and 58,450 genome-wide SNPs for FB were generated using a cost-effective genotyping-by-sequencing method (Zhao et al., 2018). Moreover, 19 of 23 randomly selected SNPs (82.60%) could effectively distinguish the 30 NB, 30 FB, and 30 F1 progenies with 100% efficiencies, indicating a high quality of the sequencing data.

Similar to SNPs, InDels are widely distributed in the genome and have been used in various biological process analyses in animals and plants (Zhang et al., 2017; Guo et al., 2019; Jain et al., 2019). However, few studies have been reported about the InDels in largemouth bass. Only a 66-bp deletion in the growth hormone releasing hormone gene was reported to be related to livability during embryonic development (Ma et al., 2014), and two InDels were identified as sex-specific markers in largemouth bass (Du et al., 2021). In this study, InDels between NB and FB were analyzed for the first time. The number of 1-3bp InDels was 160,624, making up 70.51% of all the InDels in the largemouth bass genome, and the number of InDels longer than 30 bp was 5,028. Together with the identified SNPs, our results provided useful information for the functional genomics studies for largemouth bass.

### Genetic structure analysis between NB and FB

In the past two decades, we analyzed the genetic structure of collected NB largemouth bass populations with microsatellite DNA markers, including the imported NB population in 2010, the new variety “Youlu No.1” bred in 2011, and the offspring of “Youlu No.1” bred in 2016. The *Ho* values of the three populations were 0.519, 0.480, and 0.4206, respectively; the *He* values of the three populations were 0.491, 0.454, 0.3916, respectively; the PIC values of the three populations were 0.407, 0.412, and 0.3257, respectively (Fan et al., 2019; Zhou et al., 2020). In this study, the genetic structure of NB and FB was first analyzed on the whole-genome level based on the SNPs. The population structure, PCA analysis, and LD decay rate indicated a lower genetic diversity of the NB. The nucleotide diversity (*π*) of NB was lower than those in FB, corresponding to the lower genetic diversity of the NB. In addition, the inbreeding coefficient (F) of NB was higher than FB, indicating the long-time inbreeding resulted in low genetic heterogeneities in NB.

### Application of SNP and InDel markers for breeding of largemouth bass

Because of their geographical distribution differences, it was reported that NB is more tolerant to low temperature, ammonia nitrogen, and low oxygen, while FB is more resistant to high temperatures and has a stronger stress response (Philipp et al., 1983; Fields et al., 1987; Carmichael et al., 1988; Williamson and Carmichael, 1990; Khosa et al., 2020). Abiotic stresses (e.g., heat, chilling, nutritional imbalance, and water quality) are one of the major factors which restrict the growth and development of largemouth bass (Li et al., 2020; White et al., 2020; Jia et al., 2022). Especially the high temperatures in the summer and autumn seasons, which commonly result in reduced food intake, declined growth rates and disease resistance of largemouth bass (Lin et al., 2022). In this study, through the annotation and KEGG classification of these identified SNPs and InDel markers, signal transduction, immune system, and endocrine system were found to be the top three pathways. Their results provided valuable information about the genetic mechanism of heat tolerance and stress response between the two subspecies of largemouth bass.

Through gene combination of two or more species, populations, or varieties, which have different genetic bases, hybridization alters the genetic structure of the offspring and thus increases their heterozygosity levels and genetic variation (Lippman and Zamir 2007). In this study, we detected 23 candidate SNPs and eight InDel markers among FB, NB, and their hybrids. It was noticed that most of these markers were homozygous in parents and heterozygous in the hybrids, indicating the heterozygosity levels were increased in the hybrids. Many studies have addressed the hybridization of FB and NB, while the divergence between hybrids may be advantageous in terms of their growth performance. Indeed, most experiment results demonstrated that the NB exhibited the best growth at the age of 1 year, followed by the hybrids and then the FB (Zolczynski and Davies 1976; Williamson and Carmichael 1990; Philipp and Whitt, 1991). Our previous study also indicated that the NB exhibited better growth than the hybrids. By contrast, a previous study suggested that a hybrid of NB♂ × FB♀ grows better than the two parents (Kleinsasser et al., 1990). This difference may be related to the culture environment and methods employed, as well as the different stages of fish. In terms of heat tolerance, it was reported that both the rank order of values for critical thermal maximum and chronic thermal maximum were NB♂ × FB♀> FB > NB♀ × FB♂> NB (Fields et al., 1987). Therefore, it is worth attempting to produce a hybridization progeny line, which might include the advantages in both the NB and FB. Moreover, the three InDels identified in this study will provide a PCR-based method for the germplasm identification in hybridization breeding of largemouth bass.

## Conclusion

Through whole-genome resequencing of 10 NB and 10 FB, 999,793 SNPs and 227,797 InDels were finally identified, which contributed to exploring the biological characteristic differences between NB and FB. The genetic structure analysis indicated that FB had higher genetic diversity than NB, indicating that the germplasm quality of cultured largemouth bass had markedly reduced in China. In addition, three identified InDels provided a simple PCR-based method to distinguish NB, FB, and their F1-based progenies. Our study provides an effective and accurate method for the germplasm identification of largemouth bass, which will be useful in the molecular-assisted breeding of largemouth bass in the future.

## Data Availability

The datasets presented in this study can be found in online repositories. The names of the repository/repositories and accession number(s) can be found at: https://www.ncbi.nlm.nih.gov/, PRJNA858961.
