# Supplementary material for: Genetic diversity analysis and development of molecular markers for the identification of largemouth bass (Micropterus salmoides L.) based on whole-genome re-sequencing
Source: Front Genet. 2022 Aug 29;13:936610. doi: 10.3389/fgene.2022.936610 (PMC9465168; doi:10.3389/fgene.2022.936610)
Supplement: Supplementary file 2 [file Table1.DOCX]

**Table 1 Summary of the re-sequencing results of NB and FB**

| Basic information | NB | | FB |
| --- | --- | --- | --- |
| Total Clean reads | | 619,675,540 | 633,811,124 |
| Total Clean base(bp) | | 92,025,014,273 | 94,176,077,761 |
| Length | | 150 | 150 |
| Average Q20 | | 97.52 | 97.81 |
| Average Properly mapped (%) | | 95.37% | 94.03% |
| Average Singletons mapped (%) | | 0.09% | 0.17% |
| Average Map ratio (%) | | 99.51 | 99.67 |
| Average Depth (x) | | 10.31 | 10.40 |
| Average Cover ratio (%) | | 97.33 | 95.57 |
| Average GC content (%) | | 39.99 | 40.00 |

**Table 2 Genotypes and annotation of 23 randomly selected SNPs in 30 NB, 30 FB and 30 NF individuals, respectively**

| Number/  Disruption | Genotype | | | Annotated genes or related regions |
| --- | --- | --- | --- | --- |
|  | NB (number) | FB(number) | NF(number) |  |
| SNP1/Chr1 | AA (30) | GG (30) | AG (30) | Intergenic region |
| SNP2/Chr2 | TT (30) | AA (30) | AT (30) | Son of sevenless homolog 1 |
| SNP3/Chr3 | GG (30) | AA (30) | AG (30) | Intergenic region |
| SNP4/Chr4 | GG (30) | AA (30) | AG (30) | Leucine rich repeat N-terminal domain |
| SNP5/Chr5 | TT (30) | GG (30) | GT (30) | 5-formyltetrahydrofolate cyclo-ligase |
| SNP6/Chr6 | AA (30) | CC (30) | AC (30) | Ubiquitin-conjugating enzyme E2 L3b |
| SNP7/Chr7 | TT (30) | CC (27) | GT (30) | Epidermal growth factor-like domain |
| SNP8/Chr8 | TT (28) | CC (30) | GT (30) | Nuclear apoptosis inducing factor 1 |
| SNP9/Chr9 | CC (30) | AA (30) | AC (30) | Intergenic region |
| SNP10/Chr10 | GG (30) | TT (30) | GT (30) | Prohibitin |
| SNP11/Chr11 | CC (30) | TT (30) | CT (30) | Synaptotagmin XIa |
| SNP12/Chr12 | CC (30) | TT (30) | CT (30) | Intergenic region |
| SNP13/Chr13 | GG (30) | AA (30) | AG (30) | Intergenic region |
| SNP14/Chr14 | GG (30) | AA (30) | AG (30) | Solute carrier family 35 member |
| SNP15/Chr15 | AA (30) | TT (30) | AT (30) | Peptide methionine sulphoxide reductase MsrA |
| SNP16/Chr16 | TT (30) | AA (30) | AT (30) | Intergenic region |
| SNP17/Chr17 | TT (30) | AA (30) | AT (30) | Intergenic region |
| SNP18/Chr18 | GG (30) | TT (30) | TT (30) | Intergenic region |
| SNP19/Chr19 | CC (30) | AA (30) | AC (30) | Microtubule-actin cross-linking factor 1 |
| SNP20/Chr20 | TT (28) | CC (30) | GT (30) | Holocarboxylase synthetase |
| SNP21/Chr21 | AA (30) | TT (30) | AT (30) | Intergenic region |
| SNP22/Chr22 | AA (30) | GG (30) | AG (30) | Ankyrin 3b |
| SNP23/Chr23 | AA (28) | TT (30) | AT (30) | Podocalyxin-like |

**Table 3 Primers and annotation of 8 selected InDels in largemouth bass**

| Indel  location | Insertion /  deletion size | Primer (5’-3’) | Annotated genes  or related regions | |
| --- | --- | --- | --- | --- |
| ID1/Chr17 | +100 | F: ACATTCAGCCCTCTTGACCG  R: GACACGGGGAGATCATGCAA | | Intergenic region |
| ID2/Chr2 | +90 | F: CCTTTGTTAACCTGCCCCCT  R: GTAGTCATGGGACCATCCCC | | Intergenic region |
| ID3/Chr3 | +46 | F: GCATCGTTTCCACAGGTGTC  R: GCAGCTTCCAATGCAACTGTA | | Intergenic region |
| ID4/Chr6 | +87 | F: TCACGCCACATCCAGGTAAG  R: TGCCATAGGTAACTCCCCAGT | | Intergenic region |
| ID5/Chr20 | +83 | F: CGTGTCAGCTAACTACACCTGA  R: ATACTGCCCCGCAAAGGAAA | | Intergenic region |
| ID6/Chr5 | -84 | F: GTCAACCGGTGAACACAACG  R: ACGTTATCAGCACTGTGCCA | | Neural-cadherin-like |
| ID7/Chr17 | -51 | F: AGGGAGAAACCTCATTGGGC  R: TTGCTGGCATCCTCCATAGC | | Intergenic region |
| ID8/Chr23 | -61 | F: CACCAGCCTGCAGGTAAGAA  R: CTTCCAACCACACAAGGTCAG | | Intergenic region |

Note: “+” and “-” mean insert and deletion in NB in comparison with FB, respectively.
