# Supplementary material for: Genetic diversity analysis and development of molecular markers for the identification of largemouth bass (Micropterus salmoides L.) based on whole-genome re-sequencing
Source: Front Genet. 2022 Aug 29;13:936610. doi: 10.3389/fgene.2022.936610 (PMC9465168; doi:10.3389/fgene.2022.936610)
Supplement: Supplementary file 4 [file Table2.DOCX]

**Table S1 Primers of 23 randomly detected SNPs between NB and FB.**

| SNP locus | Forward primer (5’-3’) | Reverse primer (5’-3’) | Extension primer (5’-3’) |
| --- | --- | --- | --- |
| SNP1/Chr1 | ATCTCTGGTACTGCCCTA | GGATTTCTGTTTCGTCTGAG | CTGTAAACAACCATGTCTCTTC |
| SNP2/Chr2 | TGCGATAACAGAACAGAAC | AAAGAGAAATGAAGGGACTG | ctgactgactgactgactACTGATTAGATTCATGATATTT |
| SNP3/Chr3 | AAACGGATCTTTCTGTCTCT | TTTCACGCTGCCTTATTTC | ctgaTGGAGAAGAATTTATTTGTCTC |
| SNP4/Chr4 | TCATCCAGCGAGGCAAAT | AGCCAAACCAACTAACTATCC | ctgactgactTAATTCACATTGTCTGTGTTGC |
| SNP5/Chr5 | GCCTACTACACAACCCATT | GCTTCATCCAGCAAGAGG | ctgactgactgactgactgactgactAACCCATTGTAGATACCGGAGA |
| SNP6/Chr6 | TCTTCTGCCAGTTGATGA | TGGATGTTCGCTTCACTA | ctgactgactgaTTCCTCGCCTCGTGTCTGAGGC |
| SNP7/Chr7 | CCACTTCACCTCACTGTAA | CACGATCAGAACATCAGAAC | ctgaTCTGCTGCTCCCTGAAAACAGA |
| SNP8/Chr8 | GATCAGAATCGGTGTTAGAC | TTATTATGTAACTGGAGTGAGAG | TTCTCAGCAGGAAGACTAGCTC |
| SNP9/Chr9 | TGGATGTCATACAGGACTC | TGGACACCAAATCAAAGC | ctgactgactgactgactCCTTGTTTGGGCCGGACTTTCG |
| SNP10/Chr10 | CCAGAAGTAAGGGTATTGAGA | GAGACAGCAACAGAGACA | ctgactgactgactgactgactgaGCAGTAAGTTTTTCGTTACTAC |
| SNP11/Chr11 | CCATCTGACTGTGAACTGT | GTCTGCTCTAGTGTTGCT | ctgaTGTGGCTGAAGACGAGTGGAGA |
| SNP12/Chr12 | ATTATGTGATTGAGTGCGATT | GAGAGACAGAGATGGAAAGA | ctgactgactgactATGGATGCTGTGTTTCCATTCG |
| SNP13/Chr13 | TTCAACAACATTAACATCAGGT | CCTACAGTTCATAGCGGTAT | CTGTGATATTTCTCTCACTTCC |
| SNP14/Chr14 | AATATGAAGTAGTCCAACAGAG | GATTTATCTTAACTCGTTTCTCAG | ctgactgactCAACAGAGCTAATGATGCTAAC |
| SNP15/Chr15 | TTTGTTTCCTCCTCATCTCT | AGCCCACACCATAAATACT | ctgactgactgactgactgactCTTCCTTTTCCTTGTTCCCTCC |
| SNP16/Chr16 | AAAGAAGGCACAGTATTTACC | TCCATTACTGCTGACCATT | ctgaAGCTATATTTTTCTACACTACA |
| SNP17/Chr17 | AGATCGTACTTAGTTACACAATG | TGAGGAGTGAGCAATGAG | ctgactgactgaTGAAATTCAATTAATTAATAAG |
| SNP18/Chr18 | GCTGCTGGAATGCTAATC | TGGATGAAATAAGTACAGTAGAAG | ctgactgactgactgactgactgaCAGCTAAAACAGGTTGGTTGTG |
| SNP19/Chr19 | ACAGCTTGCAGTAGTTGTAC | TTGACCTGAGAGAAACGCAG | ctgactgactgactgactCTTTATCCCACCTTCCTCCATG |
| SNP20/Chr20 | TGAAGGCACAAGTCCATT | GACTGCTGTCTTCCTCTG | ctgaCCCAGCCGACTGAAGTTATCTA |
| SNP21/Chr21 | ATATGTATTCTCCTTGCTGTTAA | TAGCCTATTTCATAAAACGAAAG | ctgactgactgaAATGCTCATTATAATAATGATT |
| SNP22/Chr22 | ACTCGACATCATCTGAATCA | ACTACAGCATCACAGCAA | TGGAGTTTATGTCGGAGTCTGT |
| SNP23/Chr23 | GAGAAGAACCGACCAGAA | GCAGAGAATCACAGTCATC | ctgactgactgactgactGTGAGTAAAAGTTAAATCAAAG |
